# Primary chondrosarcoma of the penis in a young patient: A case report and review of the literature

**DOI:** 10.1002/iju5.12230

**Published:** 2020-10-08

**Authors:** Bandar Alhubaishy, Georgios Gakis, Thomas Knoll

**Affiliations:** ^1^ Urology Department Klinikum Sindelfinge‐Boeblingen Sindelfingen Germany; ^2^ Urology Department King Abdulaziz University Hospital Jeddah Saudi Arabia; ^3^ Urology Department Wurzburg University Hospital Wuerzburg Germany

**Keywords:** chemoradiation therapy, chondrosarcoma, MRT‐pelvis, penectomy, penis‐preserving surgery

## Abstract

**Introduction:**

Primary chondrosarcoma of the penis is rare. We present a case of primary chondrosarcoma of the penis in a young patient.

**Case presentation:**

A 35‐year‐old man presented with a painless mass at the base of his penis for the past 6 months. Incisional biopsy of the lesion revealed a chondrosarcoma. Pelvic magnetic resonance imaging and computed tomography of the thorax, abdomen, and pelvis ruled out a primary lesion in the bones and soft tissues. The patient rejected total penectomy and decided to start chemoradiotherapy followed by local tumor resection.

**Conclusion:**

Primary chondrosarcoma of the penis is rare. Interdisciplinary management plays an important role in planning the therapy for rare tumors. A combined chemoradiation therapy can be followed by penis‐preserving surgery to improve the quality of life in young patients with proximal penile tumors.

**Abbreviations & Acronyms**
CT: computed tomography
ESO: extraskeletal osteosarcoma
MRI: magnetic resonance imaging
R0: negative resection margin
SCC: squamous cell carcinoma

**Keynote message**

Primary chondrosarcoma of the penis is rare. We propose a penis‐preserving approach in the treatment of a primary chondrosarcoma of the penis. This may improve the chance of an acceptable quality of life for patients with this disease.

## Introduction

Chondrosarcoma is a rare type of cancer that commonly begins in the bones but can sometimes occur in soft tissues in proximity to the bones. The incidence of chondrosarcoma is approximately 1 per 100 000 individuals worldwide.^1^ It is frequently diagnosed in patients in their fourth and fifth decades of life.^2^ It also has no racial predilection.^1^ While its pathophysiology is unknown, chromosomal anomalies in 9p21, 10, 13q14, and 17p13 have been detected in some types of chondrosarcomas.^3^

The most common primary malignant neoplasm of the penis is SCC.^4^ Mesenchymal tumors of the penis are rare, constituting <5% of all types of penile malignancies.^4^ Most often, the penile mass presents as a palpable lesion, which may be associated with penile pain, bleeding, or a foul odor.^5^ The corpora cavernosa are most commonly involved, followed by the glans and corpus spongiosum. Priapism is present in nearly 40% of cases and is more commonly associated with secondary metastasis than primary malignancies.^6^ The overall prognosis for patients presenting with ESO of the penis is poor, with a 5‐year survival rate <25%. The risk of local and distant metastases ranges from 36% to 65%.^7^

## Case presentation

**Fig. 1:**
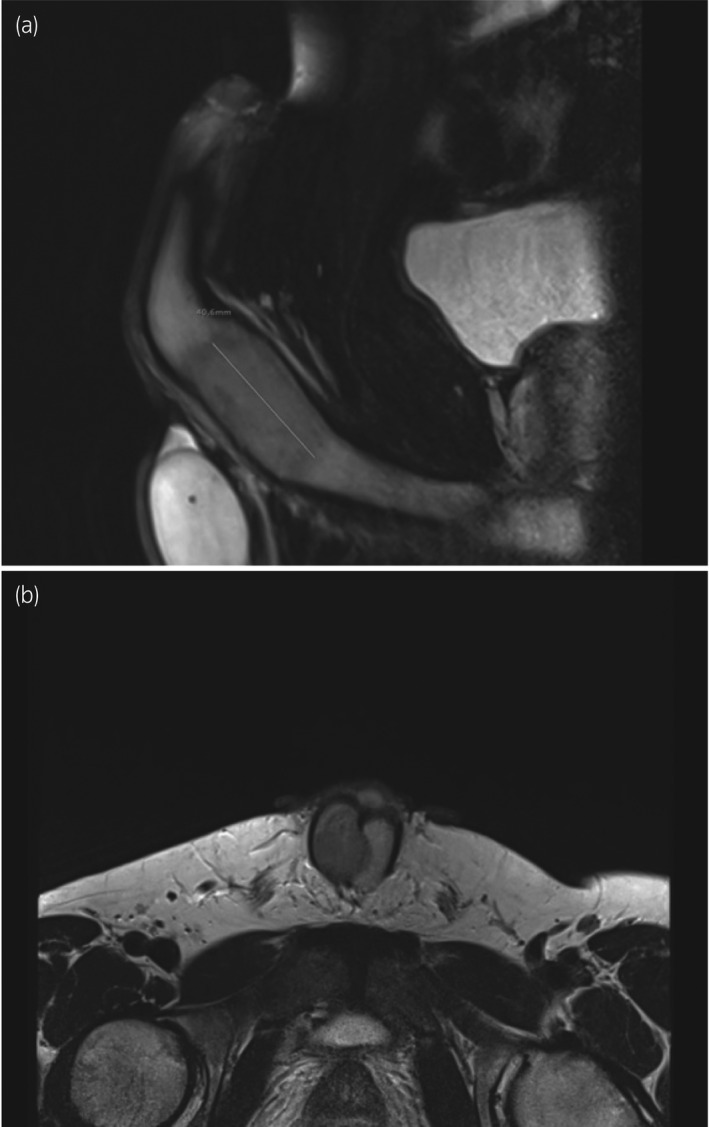
Infiltration of the right corpora cavernosa by the chondrosarcoma (3 cm) confirmed by histopathology; (a) sagittal view and (b) axial view.

**Fig. 2:**
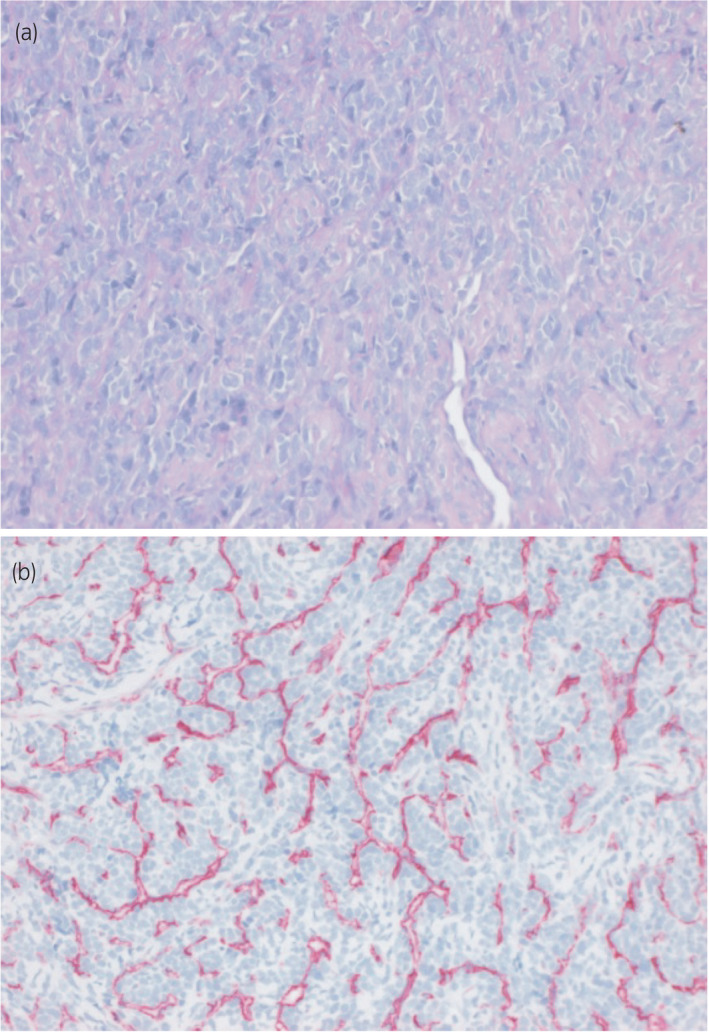
Histopathological slides of the tumor using (a) HE and (b) CD34, showing hemangiopericytoma‐like vessel structures.

**Fig. 3:**
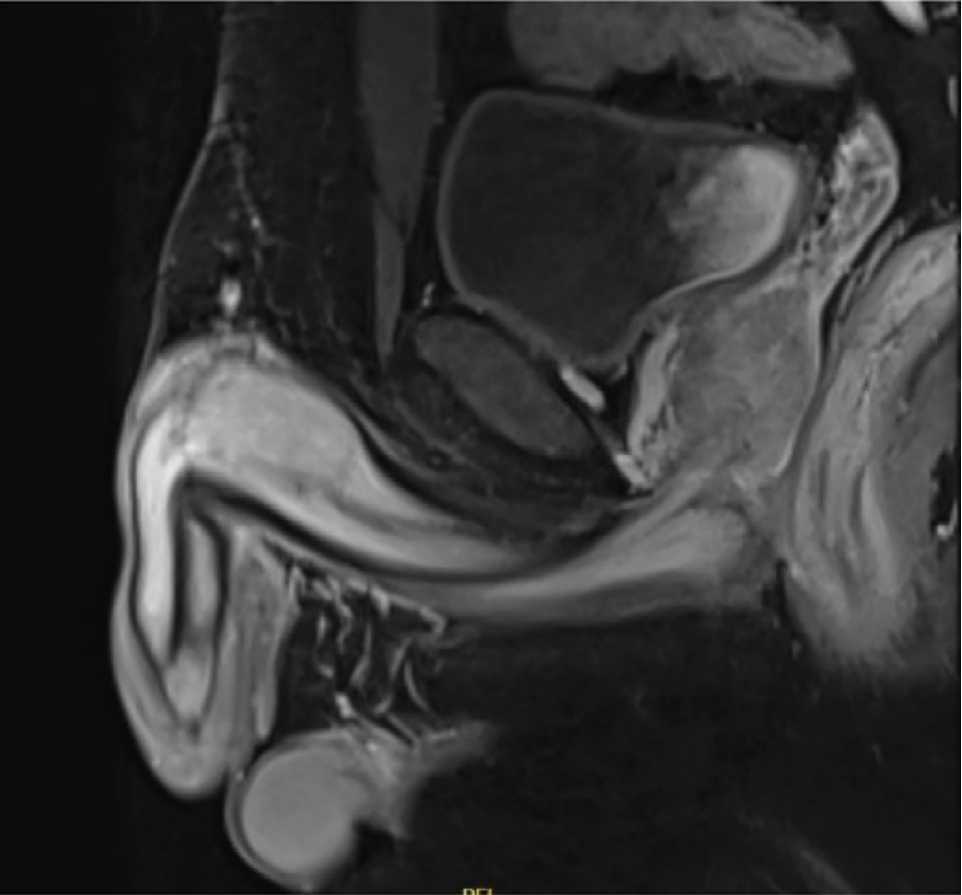
Regression of the chondrosarcoma in the right corpora cavernosa (2 cm) after chemoradiotherapy.

In January 2019, a 35‐year‐old man presented with a 6‐month history of a painless mass (3 × 2 cm) at the base of his penis. Sonographic examination revealed a hyperechoic structure with perfusion. MRI of the pelvis showed a hyperintense mass in the pelvic region without associated lymphadenopathy (Fig. 1). Suspecting a malignant lesion, incisional biopsy of the tumor was performed in January 2019. Histological examination confirmed the diagnosis of a high‐grade chondrosarcoma of the corpus cavernosa (Fig. 2). A board‐certified pathologist with expertise in sarcoma reviewed the pathology to re‐confirm the diagnosis. To rule out penile metastasis and a primary bone tumor, CT of the thorax, abdomen, and pelvis and bone scan were performed, and no primary lesion could be identified. The patient’s sexual and erectile functions were not affected. The Erection Hardness Score was 4. The inguinal lymph nodes were not enlarged on physical examination. The case was presented to the tumor board to discuss further treatment. The patient and his family were counseled extensively about the disease and treatment options, including surgical intervention, chemotherapy, and radiotherapy. Surgical intervention in the form of a total penectomy was declined by the patient because of his age. Alternatively, chemoradiotherapy followed by the resection of the tumor and implantation of a penile prosthesis was discussed. Although the risk of recurrence and of infection were considerably high, the patient opted for this treatment modality. Inductive chemotherapy was started with four cycles of doxorubicin and ifosfamide analogous to the IAWS registry protocol over 10 weeks, followed by two cycles of chemoradiotherapy over 5 weeks (5 × 1.8 Gy/week to 50.4 Gy target volume dose. 6/15 MV photons). Concurrent radiosensitizing chemotherapy was administered (ifosfamide, 3 g/m^2^). The chemotherapy was tolerated without any side effects being experienced. MRI of the pelvis after the third cycle of chemotherapy showed a regression of the primary tumor by ~1 cm (Fig. 3). Six weeks after the completion of chemoradiotherapy, a tumor resection (R0) with implantation of a penile prosthesis was carried out. During the tumor resection, three frozen sections were obtained to ensure tumor‐free resection margins (proximal nerve bundle 5 mm, distal nerve bundle 2 mm, and urethral margin 1 mm). While the pathology was consistent with the primary diagnosis of 50% regression of the tumor (vital tumor 50%), the tumor grade after chemoradiotherapy could not be identified. The patient is under regular and intense follow‐up every 3 months with MRI of the pelvis and CT of the thorax, abdomen, and pelvis every 12 months. Currently, there is neither local recurrence nor distant metastasis.

## Discussion

This case of a chondrosarcoma is unique based on its primary location. To the best of our knowledge, this is the first report in the literature of a primary chondrosarcoma of the penis. The treatment decision for our patient was based on patient desire and tumor grade. Based on the European Society for Medical Oncology guidelines, surgery is considered as a standard therapy for all patients with localized soft tissue sarcoma with wide excision and negative margins. The guidelines also indicate that neoadjuvant radiotherapy, possibly in combination with chemotherapy to a total dose of 50 Gy in 1.8–2 Gy fractions, followed by surgery, may be considered.^8^

In a single case of chondrosarcoma of the jaw that metastasized to the penis,^9^ the patient was initially treated systemically with ifosfamide. Because of local progression, radiotherapy (25 Gy) was delivered to the primary tumor, resulting in partial pain relief and a decrease in tumor volume. This patient died of disease progression 13 years after the detection of the metastatic disease.^9^

Only seven cases of primary ESO of the penis have been reported in the literature.^7^, ^10^, ^11^, ^12^, ^13^, ^14^, ^15^ Of the seven, one had areas resembling malignant fibrous histiocytoma^16^ and another had a mixed tumor with osteosarcoma and SCC. Two of these cases underwent a partial penectomy,^12^, ^14^ four underwent total penile amputation,^10^, ^11^, ^13^, ^15^ and one, which was a case with a primary ESO of the glans penis, underwent a glansectomy with glanular reconstruction.^7^

The choice of treatment modalities available is based on anecdotal information, as no prospective trials have been conducted, and no treatment has been shown to be superior in prolonging patient survival.^16^ The management of patients with this pathology should thus be individualized.

## Conclusion

This is the first report of a primary chondrosarcoma of the penis. Extensive patient counseling must be considered before treatment initiation in these cases. Inductive chemotherapy may be used to minimize the extent of surgical resection, with the aim of genital preservation. As an alternative to surgical intervention, a combination of chemotherapy and radiotherapy may be applicable. Compared with penile amputation, penis‐preserving surgery may improve patients’ quality of life, at the cost of an increased risk of local recurrence.

## Conflict of interest

The authors declare no conflict of interest.
